# 

**Published:** 1878-02

**Authors:** 


					﻿Books and Pamphlets Received.
Popular Science Monthly and Supplement.
Annual Report of Pennsylvania Free Dispensary for Skin Diseases. Phil-
adelphia: 1877.
Twenty-fifth Annual Announcement Med. Dep. Univ, of Vermont. 1878.
Annual Report of the Columbia Hospital for Women. Year ending June
30, 1877.
Dressing of Stumps, by Louis Bauer, M. D., M. R. C. S. Reprint from St.
Louis Clinical Record.
Twenty-second Annual Report of the State Lunatic Hospital at North-
ampton, for year ending Sept. 30, 1877.
fis IfffllO
Medical and Surgical Journal
PUBLISHED MONTHLY,
Containing Original Articles, Reports of Medical Societies and Hospitals, Edito-
rial, Reviews, Correspondence. Army News, etc., etc ; including the usual variety
of Medical Periodical Publications. Specimen copies sent on application.
Address Buffalo Medical & Surgical Journal, Buffalo, N. Y.
TERMS OF SUBSCRIPTION, $3.00 PER YEAR, IN ADVANCE.
AH communications for publication shoud be sent before the twentieth (20th) of each
month, or tliey will of necessity lie over until the next number. No change can be macle in
advertisements after the twenty-fifth. Address, Buffalo Medical and Surgical Journal, 978
Main Street.	I
' Correspondence and original articles are solicited. Publication is no evidence that the
views of the author arc endorsed.
				

